# Comparative Outcomes and Safety of Vedolizumab vs Tumor Necrosis Factor Antagonists for Older Adults With Inflammatory Bowel Diseases

**DOI:** 10.1001/jamanetworkopen.2022.34200

**Published:** 2022-09-30

**Authors:** Siddharth Singh, Aske T. Iversen, Kristine H. Allin, Tine Jess

**Affiliations:** 1 Division of Gastroenterology, Department of Medicine, University of California, San Diego, La Jolla; 2 Division of Biomedical Informatics, Department of Medicine, University of California, San Diego, La Jolla; 3 Center for Molecular Prediction of Inflammatory Bowel Disease, Department of Clinical Medicine, Aalborg University, Copenhagen, Denmark; 4 Department of Gastroenterology and Hepatology, Aalborg University Hospital, Aalborg, Denmark

## Abstract

**Importance:**

Observational comparative effectiveness studies can inform the positioning of biologic therapies for older patients with inflammatory bowel disease (IBD) who are underrepresented in clinical trials.

**Objective:**

To compare the effectiveness and safety of vedolizumab vs tumor necrosis factor (TNF) for older patients with IBD.

**Design, Setting, and Participants:**

This active comparator, new-user design, comparative effectiveness study was conducted between January 1, 2005, and December 31, 2018, among 754 older patients (aged ≥50 years) with IBD from the Danish National Patient Register. The mean follow-up after treatment initiation took place at 32 to 40 weeks. Statistical analysis was performed from February 1 to April 27, 2022.

**Interventions:**

Treatment with vedolizumab or TNF antagonists.

**Main Outcomes and Measures:**

The primary effectiveness outcome was treatment failure, defined as the composite risk of IBD-related hospitalization, IBD-related surgery, or a new corticosteroid prescription more than 6 weeks after initiation of treatment with biologic therapy. Secondary effectiveness outcomes were time to each individual component of the composite effectiveness outcome. The primary safety outcome was the risk of serious infections, defined as infections requiring hospitalization. A 1:1 propensity score–matched analysis was conducted, accounting for patient-, disease-, and treatment-associated factors.

**Results:**

The study compared 377 older patients with IBD with incident use of vedolizumab (202 women [53.6%]; mean [SD] age, 61.2 [8.3] years; 177 [46.9%] with Crohn disease) vs 377 patients with incident use of TNF antagonists (206 women [54.6%]; mean [SD] age, 61.3 [8.1] years; 182 [48.3%] with Crohn disease). Overall, vedolizumab was associated with an increased risk of treatment failure compared with TNF antagonists (1-year risk, 45.4% vs 34.7%; adjusted hazard ratio [HR], 1.31; 95% CI, 1.02-1.69), including higher risk of IBD-related hospitalization (1-year risk, 27.8% vs 16.3%; adjusted HR, 1.48; 95% CI, 1.03-2.15) and IBD-related major abdominal surgery (1-year risk, 21.3% vs 8.0%; adjusted HR, 2.39; 95% CI, 1.45-3.94). In subgroup analysis by IBD phenotype, among patients with Crohn disease, vedolizumab was associated with a 77% higher risk of treatment failure (adjusted HR, 1.77; 95% CI, 1.21-2.58), while no difference in risk of treatment failure was seen among patients with ulcerative colitis (adjusted HR, 1.04; 95% CI, 0.75-1.43; *P* = .03 for interaction). There was no significant difference in the risk of serious infections, overall (1-year risk, 8.2% vs 8.7%; adjusted HR, 1.04; 95% CI, 0.58-1.85) and by IBD phenotype.

**Conclusions and Relevance:**

In this comparative effectiveness study of older patients with IBD, vedolizumab was associated with a higher risk of treatment failure compared with TNF antagonists, particularly among patients with Crohn disease, without offering a significant safety advantage.

## Key Points

**Question:**

What is the comparative effectiveness and safety of vedolizumab vs tumor necrosis factor (TNF) antagonists for older patients with inflammatory bowel diseases (IBDs)?

**Findings:**

In a comparative effectiveness study of 754 older patients, vedolizumab use was associated with a higher risk of treatment failure compared with TNF antagonists, without any difference in risk of serious infections, particularly for patients with Crohn disease.

**Meaning:**

This study suggests that vedolizumab is associated with a higher risk of treatment failure compared with TNF antagonists, without offering any safety advantage for older patients with IBD.

## Teaser

This comparative effectiveness study, conducted among a Danish nationwide cohort, compares the effectiveness and safety of vedolizumab vs tumor necrosis factor for older patients with inflammatory bowel disease (IBD).

## Introduction

The incidence, prevalence, and health care costs of inflammatory bowel disease (IBD) among older adults are rapidly increasing relative to younger adults, with approximately 1 in 3 patients with IBD expected to be older.^1^ However, there is a paucity of evidence-based treatment guidance for older patients with IBD, who represent less than 5% of participants in IBD clinical trials, leading to substantial practice variability and inferior outcomes.^2^ The risk of disease-related complications in older patients is underappreciated: risk of surgery, hospitalization, and corticosteroid treatment are comparable in older vs younger patients.^3^ However, older patients are frequently undertreated and mismanaged with long-term corticosteroid use and limited use of steroid-sparing therapies owing to patients’ and clinicians’ concerns about the safety of immunosuppressive therapy, which is associated with increased morbidity and mortality.^4^ There is considerable need for evidence-based treatment guidance for older patients with IBD.

During the past 2 decades, the therapeutic armamentarium for the medical management of patients with moderate-to-severe IBD has substantially expanded.^5^ Although tumor necrosis factor (TNF) antagonists have been the mainstay of treatment for patients with IBD refractory to conventional therapies, newer therapies such as vedolizumab, an anti-integrin monoclonal antibody, potentially offers a safety advantage because of its gut selectivity, which may be attractive for older patients. With a paucity of head-to-head comparisons and low representation of older patients with multiple comorbidities in clinical trials, observational studies on the comparative effectiveness and safety of different therapies can inform routine clinical practice in this understudied but increasingly prevalent and vulnerable older population. Hence, we conducted a nationwide propensity score–matched cohort study comparing the effectiveness and safety of vedolizumab vs TNF antagonists for older patients with IBD in Denmark.

## Methods

This study was approved by the Danish Data Protection Agency. Ethics approval is not required for registry-based research in Denmark. Patient consent is also waived for registry-based research by the Danish Data Protection Agency. All data were deidentified. We followed the International Society for Pharmacoeconomics and Outcomes Research (ISPOR) reporting guideline for comparative effectiveness research.^6^

### Data Source and Study Population

The source population consisted of all individuals aged 50 years or older and living in Denmark between January 1, 2005, and December 31, 2018, according to the Danish Civil Registration System. Using the unique personal identification number given to each Danish citizen at birth, the population was linked to the Danish National Patient Registry, which contains information on all hospitalizations in Denmark since 1977 and all outpatient visits and emergency department contacts since 1995. In the Danish National Patient Registry, we identified (1) older adults; (2) with an IBD diagnosis at age 50 years or older (≥1 registration with an IBD diagnosis), based on *International Classification of Diseases, Eigth Revision* (*ICD-8*) code 563.01-09 or *International Statistical Classification of Diseases and Related Health Problems, Tenth Revision* (*ICD-10*) code K50 for Crohn disease (CD) or *ICD-8* code 563.19 or 569.04 or *ICD-10* code K51 for ulcerative colitis (UC); (3) who were treated with TNF antagonists or vedolizumab after IBD diagnosis; and (4) who had lived in Denmark at least 5 years prior to treatment initiation. Using a pathology database as reference, an assessment of nearly 800 patients estimated the completeness of registration of IBD in the Danish National Patient Registry to be 94%, whereas the estimated validity, expressed as the proportion of confirmed diagnoses in the registry, was 97% for CD and 90% for UC.^7^

Information on prescription of TNF antagonists (infliximab, adalimumab, and golimumab) and vedolizumab was obtained using procedure codes from the Danish National Prescription Register. Although treatment with TNF antagonists for IBD was introduced in Denmark in 1999, we started the study in 2005; in this way, patients who were early users of biologic therapy who may have been treated in the first years after the introduction of TNF antagonists (who are likely to be different from the drugs’ eventual stable user population, in terms of factors such as disease severity, and therefore may introduce bias) were excluded. In case a patient received diagnosis codes for both UC and CD, the most frequent diagnosis code was used to assign IBD phenotype.

### Exposure and Comparator

The primary exposure of interest was treatment with vedolizumab, and the primary comparator was treatment with TNF antagonists. We considered patients as being continuously exposed from the index date (date of first registration of a biologic agent) for the duration of their prescription. We restricted exposure to incident use of infliximab, adalimumab, golimumab, and vedolizumab. However, a patient could be an incident user of multiple different biologic agents and contribute exposure time to different groups of biologic agents at different times. We pooled treatment episodes of different TNF antagonists into one comparator group. Patients were followed up until occurrence of the outcome of interest, treatment discontinuation (absence of new registration of therapeutic agent for >4 months), switch to alternative biologic treatment, emigration, death, or completion of the study (last date of follow-up, December 31, 2018).

### Outcomes

#### Effectiveness

The primary effectiveness outcome was treatment failure, defined as a composite of time to IBD-related hospitalization (IBD as primary discharge diagnosis), IBD-related major abdominal surgery (including intestinal resection, colectomy, and stoma creation),^8^ or new corticosteroid prescription more than 6 weeks after biologic therapy initiation. Secondary effectiveness outcomes were time to each individual component of the composite effectiveness outcome.

#### Safety

The primary safety outcome was risk of serious infections (defined as infections requiring hospitalization, based on *ICD-10* diagnosis codes of infections of the respiratory tract, skin and soft tissue, genitourinary tract, gastrointestinal tract, central nervous system, and septicemia or sepsis).^9^ Secondary safety outcomes were risk of cancer (solid-organ cancer, hematologic cancer, melanoma)^10^ and major adverse cardiovascular events (MACE) and/or venous thromboembolic events.^11,12^

### Covariates

We collected baseline covariates (at time of start of biologic therapy or in the preceding 12 months), including (1) demographic characteristics (age at time of biologic therapy initiation, sex, socioeconomic status); (2) disease and treatment characteristics, including IBD phenotype (CD or UC), disease duration, prior TNF antagonist prescription and response to prior TNF antagonists (no prior exposure, primary nonresponse, secondary loss of response in the preceding 12 months), prior exposure to corticosteroids and thiopurines (primarily azathioprine, which accounts for >99% of thiopurine prescriptions in Denmark) in the baseline 6 months or less prior to biologic therapy initiation, concomitant treatment with immunomodulators (prescription within 0-3 months after biologic therapy initiation); and (3) health care use, including comorbidity burden measured by the Charlson Comorbidity Index (CCI) score, frailty status (based on Hospital Frailty Risk score), IBD-related major abdominal surgery (all examined within ≤5 years prior to exposure to biologic therapy), IBD-related hospitalization, and serious infection (within ≤12 months prior to biologic therapy exposure).^13,14^ We did not have access to individual participants’ medical records, endoscopy reports, or biochemical parameters.

### Statistical Analysis

Statistical analysis was performed from February 1 to April 27, 2022. To compare vedolizumab vs TNF antagonists, we performed 1:1 propensity score–matched analysis (primary analysis), without replacement to account for differences in baseline covariates. The propensity score model included demographic variables, disease and treatment characteristics, comorbidity burden, and health care use, as outlined previously. We measured the standardized difference of each covariate in the propensity score model, and variables were considered to be different across treatment if, after propensity score matching, the standardized difference was greater than 10%. To correct for remaining imbalance after propensity score matching was performed, we included remaining covariates that were shown to be different across treatment groups into the final multivariate Cox proportional hazards regression models for assessment of the outcomes of interest. We performed secondary analysis using the inverse probability of treatment weight (IPTW) approach.^15^ The IPTW analysis was derived by using the propensity score on all observations before matching. In contrast to propensity score matching, in which the sample size usually decreases (as a result of matching), this type of modeling allowed us to retain all identified patients in the analysis, resulting in increased power.

We performed preplanned subgroup analysis, comparing the effectiveness and safety of vedolizumab vs TNF antagonists, based on age at time of initiation of biologic therapy (50-60 years vs >60 years), sex (male vs female), IBD phenotype (CD vs UC), and whether patients were treated with biologic monotherapy vs combination treatment with immunomodulators. We hypothesized a priori that vedolizumab might be associated with higher risk of treatment failure than TNF antagonists among patients with CD, but not among patients with UC, with no significant differences in the risk of serious infections. We hypothesized that the effectiveness and safety of vedolizumab and TNF antagonists would not be different in other stratified analyses. After peer review, we performed additional post hoc subgroup analyses based on alternative age categories (50-70 years vs >70 years) and burden of comorbidities (CCI score of 0 vs ≥1).

We estimated hazard ratios (HRs) with 95% CIs using a robust sandwich estimate to account for the dependency in the matched pairs.^16^ Hazard ratios were estimated for each outcome of interest separately using time since biologic therapy initiation as the underlying time scale, and patients were censored at time of treatment discontinuation, treatment switch, death, emigration, or end of observation period (December 31, 2018). Because we censored at treatment discontinuation and treatment switch, there is a possibility for dependent censoring. We used the method described by Lee and Wolfe^17^ to test if the censoring was independent of each of the 3 outcomes, IBD-related hospitalization, corticosteroid use, and IBD-related major abdominal surgery. From the tests we concluded that the censoring mechanism could be assumed to be independent of the outcomes. All *P* values were from 2-sided tests, and the results were deemed statistically significant at *P* < .05. All statistical analyses were performed using SAS software, version 9.4 (SAS Institute Inc).

## Results

### Cohort Characteristics

We compared 377 incident users of vedolizumab (202 women [53.6%]; mean [SD] age, 61.2 [8.3] years; 177 [46.9%] with CD) with 377 incident users of specific TNF antagonists (206 women [54.6%]; mean [SD] age, 61.3 [8.1] years; 182 [48.3%] with CD) after 1:1 propensity score matching (Table 1; the eFigure in the Supplement shows covariate balance plots before and after matching). A total of 38 patients treated with vedolizumab (10.1%) and 100 patients treated with TNF antagonists (25.6%) were naive to all biologic therapies. The mean (SD) follow-up after starting therapy varied by outcome and ranged from 33 (30) to 40 (31) weeks among patients treated with vedolizumab and from 32 (30) to 39 (32) weeks among patients treated with TNF antagonists.

**Table 1.** Baseline Characteristics of Older Patients With IBD Treated With Vedolizumab vs TNF Antagonists, After 1:1 Propensity Score Matching^a^

Baseline characteristic	No. (%)	
	Incident users of vedolizumab (n = 377)	Incident users of specific TNF antagonists (n = 377)
Age of patients, y		
50-60	196 (52.0)	193 (51.2)
61-70	109 (28.9)	116 (30.8)
>70	72 (19.1)	68 (18.0)
Sex		
Female	202 (53.6)	206 (54.6)
Male	175 (46.4)	171 (45.4)
Area socioeconomic index, quartile		
1	95 (25.2)	83 (22.0)
2	96 (25.5)	91 (24.1)
3	118 (31.3)	122 (32.4)
4	68 (18.0)	81 (21.5)
IBD subtype		
Crohn disease	177 (46.9)	182 (48.3)
Ulcerative colitis	200 (53.1)	195 (51.7)
Follow-up, mean (SD), mo^b^	7.6 (7.0)	7.6 (7.4)
Disease duration, mean (SD), y	12.0 (10.5)	12.5 (10.2)
Charlson Comorbidity Index score		
0	249 (66.0)	257 (68.2)
1	67 (17.8)	67 (17.8)
≥2	61 (16.2)	53 (14.1)
Hospital frailty risk score		
Low risk (<5)	340 (90.2)	343 (91.0)
Intermediate risk (5-15)	27 (7.2)	27 (7.2)
High risk (>15)	10 (2.7)	7 (1.9)
Disease characteristics		
IBD hospitalization within 1 y prior to biologic therapy initiation	138 (36.7)	116 (30.8)
IBD-related major surgery within 5 y prior to biologic therapy initiation	52 (13.8)	58 (15.4)
IBD-related minor surgery within 5 y prior to biologic therapy initiation	20 (5.3)	26 (6.8)
Serious infection within 1 y prior to biologic therapy initiation	41 (10.9)	32 (8.5)
Treatment characteristics		
Concomitant immunomodulator use with biologic therapy initiation	25 (6.7)	48 (12.8)
Concomitant corticosteroid use with biologic therapy initiation	122 (32.5)	78 (20.8)
Azathioprine use ≤6 mo prior to biologic therapy initiation	57 (15.1)	46 (12.2)
Corticosteroid use ≤6 mo prior to biologic therapy initiation	173 (45.9)	157 (41.6)
TNF antagonist exposure ≤12 mo prior to index biologic therapy initiation		
None	110 (29.2)	107 (28.4)
Primary nonresponse to TNF antagonist	42 (11.1)	49 (13.0)
Secondary loss of response to TNF antagonist	224 (59.7)	221 (58.6)

Abbreviations: IBD, inflammatory bowel disease; TNF, tumor necrosis factor.

^a^ Patients could contribute to multiple different exposures; hence, the unit of analysis was patient-treatment episode.

^b^ Patients were censored at time of primary effectiveness or safety outcome; follow-up varied by outcome.

These patients were identified from a cohort of 39 207 patients with IBD aged 50 years or older in Denmark between 2005 and 2018. Of these patients, we identified 3132 incident users of specific TNF antagonists (2175 infliximab, 779 adalimumab, and 178 golimumab) and 379 incident users of vedolizumab (eTable 1 in the Supplement). In the overall cohort, 365 patients (48.4%) were older than 60 years at time of biologic therapy initiation; 177 patients receiving vedolizumab (46.9%) and 182 patients receiving TNF antagonists (48.3%) had CD (Table 1). Patients treated with vedolizumab were more likely than those treated with TNF antagonists to have multimorbidity (CCI score ≥2, 61 [16.2%] vs 53 [14.1%]) and a higher burden of frailty (high risk score, 10 [2.7%] vs 7 [1.9%]). No significant differences were observed in the proportion of patients with recent immunomodulator and corticosteroid exposure.

### Comparative Effectiveness of Vedolizumab vs TNF Antagonists

**Figure 1.**
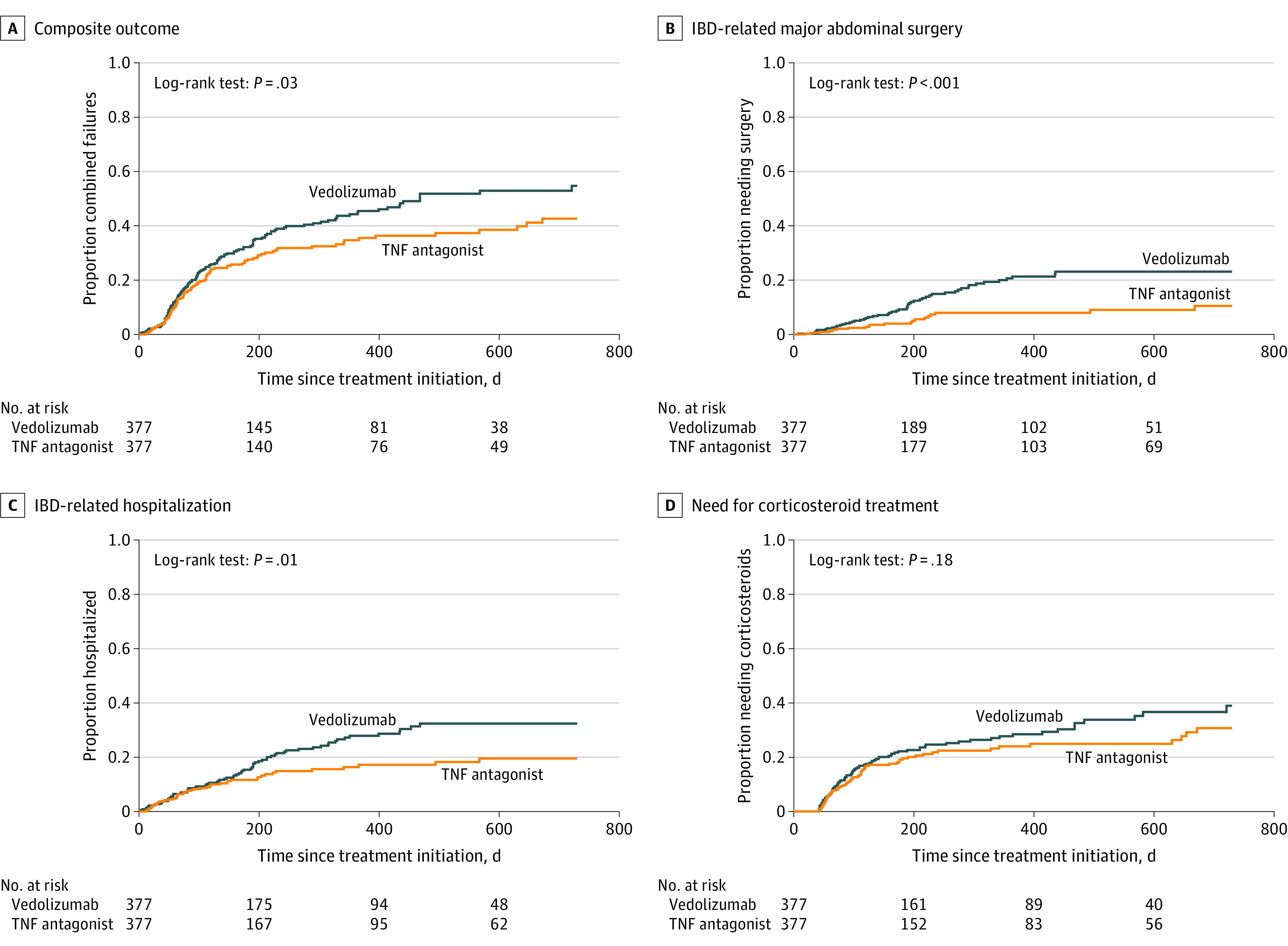
Cumulative Risk of Outcomes Among Older Patients With Inflammatory Bowel Disease (IBD) Treated With Vedolizumab vs Tumor Necrosis Factor (TNF) Antagonists in the Danish Nationwide Registry

**Figure 2.**
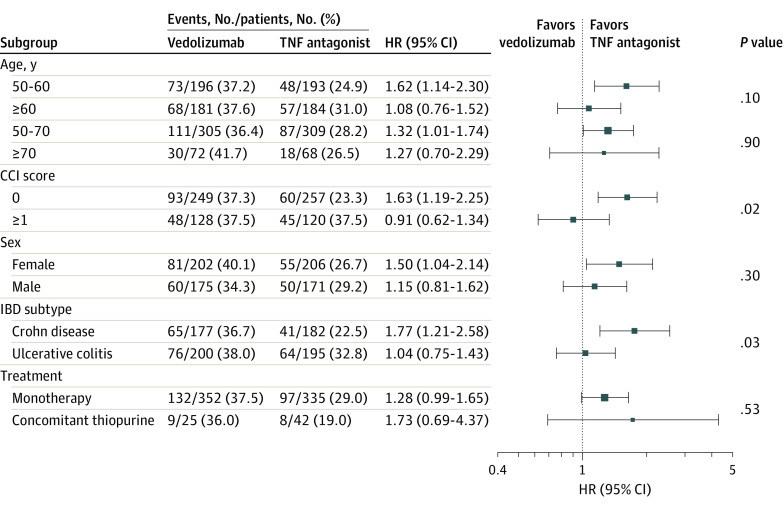
Subgroup Analysis Comparing Risk of Adverse Composite Effectiveness Outcome Among Older Patients With Inflammatory Bowel Disease (IBD) Treated With Vedolizumab vs Tumor Necrosis Factor (TNF) Antagonists The squares indicate the summary hazard ratio (HR), and the whiskers indicate the 95% CI for that specific category of the subgroup; *P* values are for interaction between subgroups. CCI indicates Charlson Comorbidity Index.

Overall, vedolizumab was associated with a 31% higher risk of treatment failure compared with TNF antagonists in the 1:1 propensity score-matched cohort (1-year risk, 45.4% vs 34.7%; adjusted HR, 1.31; 95% CI, 1.02-1.69) (Figure 1; Table 2). In subgroup analysis by IBD phenotype, vedolizumab was associated with a 77% higher risk of experiencing treatment failure vs TNF antagonists among patients with CD (adjusted HR, 1.77; 95% CI, 1.21-2.58), whereas no significant differences in the risk of treatment failure were observed among patients with UC (adjusted HR, 1.04; 95% CI, 0.75-1.43; *P* = .03 for interaction) (Figure 2; Table 2). Results were stable in subgroup analysis by age at time of biologic therapy initiation (50-60 vs >60 years; and post-hoc age groups of 50-70 vs >70 years), sex (male vs female), and whether patients were treated with biologic monotherapy vs combination therapy with immunomodulators (Figure 2). In post hoc subgroup analysis based on CCI score, treatment with vedolizumab was associated with a 63% higher risk of treatment failure vs TNF antagonists (adjusted HR, 1.63; 95% CI, 1.19-2.25) only for patients without comorbidities (CCI score 0), but not for patients with comorbidities (adjusted HR, 0.91; 95% CI, 0.62-1.34; *P* = .02 for interaction).

**Table 2.** Comparative Effectiveness and Safety of Vedolizumab vs TNF Antagonists Among Older Patients With IBD, Using Propensity Score Matched Analysis

Outcome	Vedolizumab		TNF antagonists		Vedolizumab vs TNF antagonists, HR (95% CI)
	No. of events	Incidence rate, per 100 person-years	No. of events	Incidence rate, per 100 person-years	
**IBD**					
Effectiveness outcomes					
Composite treatment failure^a^	141	59	105	44	1.31 (1.02-1.69)^b^
IBD-related hospitalization	77	28	48	17	1.48 (1.03-2.15)^b^
IBD-related major abdominal surgery	53	18	21	7	2.39 (1.45-3.94)^b^
New corticosteroid use	89	35	71	28	1.24 (0.91-1.68)^b^
Safety outcomes					
Serious infection	26	9.0	24	8.4	1.04 (0.58-1.85)
Major adverse cardiovascular events	12	4.1	7	2.4	1.68 (0.68-4.16)
**Crohn disease**					
Effectiveness outcomes					
Composite treatment failure^a^	65	60	41	31	1.77 (1.21-2.58)
IBD-related hospitalization	32	26	25	17	1.36 (0.81-2.30)
IBD-related major abdominal surgery	22	17	10	7	2.37 (1.15-4.90)
New corticosteroid use	42	36	22	15	2.14 (1.29-3.55)
Safety outcomes					
Serious infection	12	9	11	7	1.17 (0.51-2.70)
**Ulcerative colitis**					
Effectiveness outcomes					
Composite treatment failure^a^	76	58	64	61	1.04 (0.75-1.43)
IBD-related hospitalization	45	30	23	18	1.75 (1.06-2.89)
IBD-related major abdominal surgery	31	19	11	8	2.42 (1.25-4.68)
New corticosteroid use	47	34	49	43	0.83 (0.56-1.24)
Safety outcomes					
Serious infection	14	9	13	10	0.93 (0.43-1.99)

Abbreviations: HR, hazard ratio; IBD, inflammatory bowel disease; TNF, tumor necrosis factor.

^a^ Composite treatment failure was defined as a composite of time to IBD-related hospitalization (IBD as primary discharge diagnosis), IBD-related major abdominal surgery (including intestinal resection, colectomy, and stoma creation), or new corticosteroid prescription more than 6 weeks after biologic therapy initiation.

^b^ Additionally adjusted for IBD-related hospitalization in the preceding 1 year.

On examination of each effectiveness outcome individually, vedolizumab was associated with a higher risk of IBD-related hospitalization (1-year risk, 27.8% vs 16.3%; adjusted HR, 1.48; 95% CI, 1.03-2.15) and IBD-related major abdominal surgery (1-year risk, 21.3% vs 8.0%; adjusted HR, 2.39; 95% CI, 1.45-3.94) compared with TNF antagonists (Table 2). No statistically significant difference was observed in the need for corticosteroids with vedolizumab vs TNF antagonists (1-year risk, 28.4% vs 24.0%; adjusted HR, 1.24; 95 CI, 0.91-1.68). In subgroup analysis, vedolizumab was associated with a greater need for corticosteroids compared with TNF antagonists among patients with CD (adjusted HR, 2.14; 95% CI, 1.29-3.55), with no significant differences observed among patients with UC (adjusted HR, 0.83; 95% CI, 0.56-1.24; *P* = .005 for interaction). Overall findings were similar in sensitivity analysis using the IPTW approach for analysis, retaining all patients (eTable 2 in the Supplement).

### Comparative Safety of Vedolizumab vs TNF Antagonists

**Figure 3.**
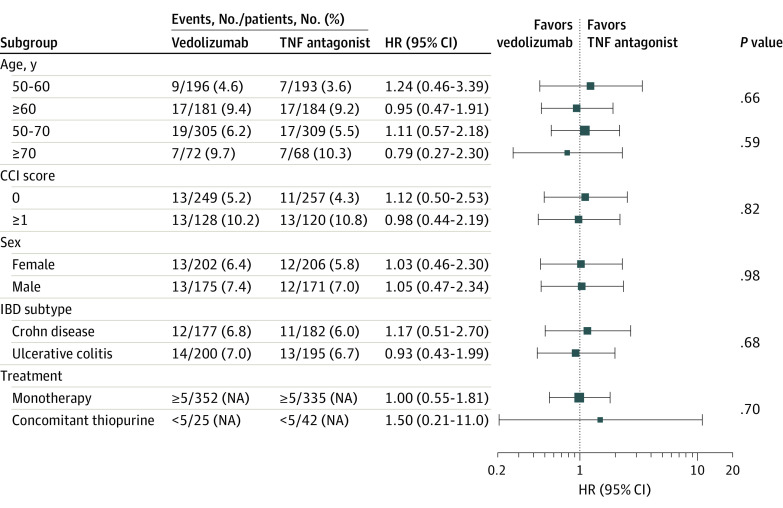
Subgroup Analysis Comparing Risk of Serious Infections Among Older Patients With Inflammatory Bowel Disease (IBD) Treated With Vedolizumab vs Tumor Necrosis Factor (TNF) Antagonists The squares indicate the summary hazard ratio (HR), and the whiskers indicate the 95% CI for that specific category of the subgroup; *P* values are for interaction between subgroups. CCI indicates Charlson Comorbidity Index; and NA, not applicable.

Overall, we did not observe any significant differences in the risk of serious infections between patients treated with vedolizumab and patients treated with TNF antagonists in the 1:1 propensity score–matched cohort (1-year risk, 8.2% vs 8.7%; adjusted HR, 1.04; 95% CI, 0.58-1.85) (Table 2). In subgroup analysis, no significant differences were observed between vedolizumab vs TNF antagonists, based on IBD phenotype (CD: adjusted HR, 1.17; 95% CI, 0.51-2.70; UC: adjusted HR, 0.93; 95% CI, 0.43-1.99; *P* = .68 for interaction), age at time of biologic therapy initiation (50-60 years: adjusted HR, 1.24; 95% CI, 0.46-3.39; >60 years: adjusted HR, 0.95; 95% CI, 0.47-1.91; *P* = .66 for interaction), sex (male: adjusted HR, 1.05; HR, 0.47-2.34; female: adjusted HR, 1.03; 95% CI, 0.46-2.30; *P* = .98 for interaction), and whether patients were treated with biologic monotherapy vs combination therapy with immunomodulators (monotherapy: adjusted HR, 1.00; 95% CI, 0.55-1.81; combination therapy: adjusted HR, 1.50; 95% CI, 0.21-11.0; *P* = .70 for interaction) (Figure 3). These findings were also observed in additional post hoc subgroup analyses based on alternative age groups (50-70 years: adjusted HR, 1.11; 95% CI, 0.57-2.18 >70 years: adjusted HR, 0.79; 95% CI, 0.27-2.30; *P* = .59 for interaction) and burden of comorbidities (CCI score 0: adjusted HR, 1.12; 95% CI, 0.50-2.53; CCI score ≥1: adjusted HR, 0.98; 95% CI, 0.44-2.19; *P* = .82).

The overall incidence of MACE and venous thromboembolic events was similar among patients with IBD treated with vedolizumab vs TNF antagonists (1-year risk of 4.0% vs 2.8%; adjusted HR, 0.90; 95% CI, 0.41-2.01). Rates of new malignant neoplasms were very low (<5 events) and could not be reported per Danish registry reporting rules.

## Discussion

In a nationwide propensity score–matched comparative effectiveness study, we made several key observations on the effectiveness and safety of vedolizumab vs TNF antagonists for older patients with IBD. First, we observed that vedolizumab was associated with a higher risk of treatment failure compared with TNF antagonists for older patients with CD, with no significant difference for patients with UC. Vedolizumab was associated with a higher risk of hospitalization due to IBD and undergoing IBD-related major abdominal surgery. No significant differences were observed in other subgroups based on age at time of biologic therapy initiation, sex, and use of biologic monotherapy vs combination therapy with immunomodulators. Second, the overall risk of serious infections, MACE, and venous thromboembolic events was comparable among patients treated with vedolizumab vs TNF antagonists. These findings provide robust evidence on the comparative effectiveness and safety of vedolizumab vs TNF antagonists among older patients with IBD and can directly inform decision-making.

There is a paucity of head-to-head clinical trials in IBD. Hence, most data on comparative effectiveness and safety of therapies are derived from network meta-analyses or observational comparative effectiveness studies. Network meta-analyses in IBD suggest that TNF antagonists may be the most efficacious for management of moderate to severe CD, whereas vedolizumab and infliximab may be equally efficacious for patients with moderate to severe UC.^18,19^ However, older patients are underrepresented in these clinical trials. There have been limited observational studies comparing the effectiveness and safety of vedolizumab vs TNF antagonists. In a retrospective cohort study using an active comparator, new-user design in the US Medicare database of adults aged 65 years and older, Kochar and colleagues^20^ compared 480 patients treated with vedolizumab vs 1152 patients treated with TNF antagonists, using propensity score weighted analysis. Patients treated with TNF antagonists were more likely to have received corticosteroids prior to and concomitantly with initiation of biologic therapy. They observed no significant differences in the crude incidence rates of IBD-related hospitalization, IBD-related surgery, and new corticosteroid use between patients treated with vedolizumab vs those treated with TNF antagonists, among all patients with IBD, or among subgroups of patients with CD or UC. Their observations are in contrast to findings in our study. We observed a significantly higher incidence of IBD-related hospitalization and IBD-related major abdominal surgery among our cohort, which may be associated with differences in patient characteristics and treatment approaches in Denmark vs the US, or differences in the accuracy of coding and definitions for IBD-related hospitalization and surgery. We classified older patients as age older than 50 years compared with age older than 65 years in the study by Kochar and colleagues^20^; however, our findings were stable in subgroup analysis by age. Cumulative 1-year risk of IBD-related hospitalization in population-based inception cohorts of older adults have been 20% to 40%,^3,21^ more consistent with findings in our cohort, and significantly higher than rates observed by Kochar et al.^20^ Similarly, the rate of IBD-related surgery is approximately 10% to 15% among the general population of older patients, with conceivably higher rates among patients with moderate to severe disease requiring biologic therapy.^22^ In a Veterans Affairs cohort of older patients with IBD, Khan and colleagues^23^ observed that the 1-year cumulative risk of IBD-related hospitalization and IBD-related surgery among patients treated with vedolizumab was 11.3% and 3.9%, respectively.

Vedolizumab is believed to be a safer medication than TNF antagonists, with a lower burden of systemic immune suppression. Vedolizumab’s gut specificity was confirmed in a vaccination study in healthy volunteers, in which it selectively reduced response to orally administered antigens, but not to parenterally administered antigens.^24^ However, 2 key factors determine the safety of biologic therapy for patients with IBD. First, the intrinsic immunosuppressive effect of the agent, and second, its effectiveness in controlling disease, achieving corticosteroid-free remission, and avoiding disease-related complications. Prior large database studies have observed that, overall, there may be no significant differences in the risk of serious infections between patients with IBD who are treated with vedolizumab and those treated with TNF antagonists.^11,25^ However, vedolizumab was associated with a lower risk of serious infections compared with TNF antagonists among patients with UC, with no significant difference observed among patients with CD. Moreover, vedolizumab was associated with a higher risk of serious gastrointestinal infections, particularly among patients with CD, including *Clostridioides difficile* colitis and infectious complications related to penetrating and/or perianal CD. In these studies, overall findings were similar in older and younger patients. In contrast, Kochar and colleagues^20^ observed that vedolizumab may be associated with a lower risk of serious infections compared with TNF antagonists, with no significant differences between patients with CD and UC. However, they excluded patients who may be experiencing infectious complications related to penetrating and/or perianal CD, which may be associated with inadequate disease control. In registry studies,^26,27^ these potentially disease-related infections account for nearly half the serious infections observed. This may explain the lower overall incidence of serious infections in their cohort. In our nationwide cohort, similar to prior findings, we observed no significant differences in the risk of serious infections among older patients treated with vedolizumab vs TNF antagonists.

### Limitations

Although we adopted a meticulous approach, using a robust nationwide registry capturing diagnoses of IBD and incident users of biologic agents in the Danish population, and applied robust propensity score methods models to account for treatment selection and a priori defined subgroup analyses, we acknowledge several important limitations to our study. First, as a nationwide register-based study, we did not have access to subjective or objective measures of disease activity or endoscopy reports and did not have accurate details of disease location or extent, behavior, and whether treatment was escalated or optimized based on drug concentration. However, our measurement of treatment exposure and outcomes was robust, but there may have been slight variability in the timing of medication as expected in routine clinical care. Second, as with any observational study, we cannot rule out unobserved confounders, especially those owing to treatment selection; however, our analytical approach, with an incident user design, accounting for key patient-, disease-, and treatment-associated covariates, including corticosteroid exposure that may serve as a surrogate of disease activity, provides some protection against bias. Results of subgroup analyses should be interpreted with caution because the remaining covariates in the propensity score may not be fully balanced. Third, we defined older patients as those older than 50 years, compared with prior studies that have variably defined older patients as older than 60 to 65 years. However, subgroup analyses based on 2 different age group exposures at the time of biologic therapy initiation did not demonstrate significant differences in findings. Recent studies have suggested that the burden of comorbidities and frailty may be more relevant factors associated with adverse outcomes than chronological age; we accounted for these key factors in our analyses.^28,29,30^ Fourth, owing to low event rate, we were unable to compare other safety outcomes such as risk of MACE, venous thromboembolic events, and cancer. We opted to focus on serious infections, defined as infections requiring hospitalization, rather than capturing all infections. Ideally, infections would be adjudicated by medical record review and microbiology data, but this level of data was not available to us. However, our definition of serious infections requiring hospitalization has been validated with a high positive predictive value.^31^

## Conclusions

In this nationwide propensity score–matched comparative effectiveness study, vedolizumab was associated with a higher risk of treatment failure compared with TNF antagonists, particularly among patients with CD, with no differences in the risk of serious infections. In the absence of predictive biomarkers, these findings suggest that older patients with CD, particularly those at higher risk of disease-associated complications, may be preferentially treated with TNF antagonists rather than vedolizumab. Future prospective registry and observational studies are warranted to confirm these findings and evaluate the comparative effectiveness and safety of other non-TNF antagonist biologic therapies such as ustekinumab and Janus kinase inhibitors. The interplay of effectiveness and relative safety of different agents, among patients who respond vs those who do not respond to therapy, also merits close evaluation to understand risk-benefit tradeoffs of novel therapies. These findings will inform optimal choice of different biologic agents depending on a patient’s risk of disease- and treatment-associated complications.

## Notes

Supplement. **eTable 1.** Baseline Characteristics of Older Patients With IBD Treated With Vedolizumab vs TNF Antagonists Before Propensity Score Matching **eTable 2.** Comparative Effectiveness and Safety of Vedolizumab vs TNF Antagonists in Older Patients With IBD, Using Inverse Probability of Treatment Weighted Analysis **eFigure.** Standardized Mean Differences of Covariates Before and After Propensity Score Matching

## References

[1] Coward S, Clement F, Benchimol EI, . Past and future burden of inflammatory bowel diseases based on modeling of population-based data. Gastroenterology. 2019;156(5):1345-1353.e4. doi:10.1053/j.gastro.2019.01.002 30639677

[2] Rabinowitz LG, Rabinowitz DG, Silver EM, Oxentenko AS, Williams KE, Silver JK. Disparities persist in inclusion of female, pregnant, lactating, and older individuals in inflammatory bowel disease clinical trials. Gastroenterology. 2022;163(1):8-13. doi:10.1053/j.gastro.2022.03.016 35288114

[3] Rozich JJ, Dulai PS, Fumery M, Sandborn WJ, Singh S. Progression of elderly onset inflammatory bowel diseases: a systematic review and meta-analysis of population-based cohort studies. Clin Gastroenterol Hepatol. 2020;18(11):2437-2447.e6. doi:10.1016/j.cgh.2020.02.048 32142940 PMC7490750

[4] Govani SM, Wiitala WL, Stidham RW, . Age disparities in the use of steroid-sparing therapy for inflammatory bowel disease. Inflamm Bowel Dis. 2016;22(8):1923-1928. doi:10.1097/MIB.0000000000000817 27416039 PMC4956567

[5] Baumgart DC, Le Berre C. Newer biologic and small-molecule therapies for inflammatory bowel disease. N Engl J Med. 2021;385(14):1302-1315. doi:10.1056/NEJMra1907607 34587387

[6] Berger ML, Mamdani M, Atkins D, Johnson ML. Good research practices for comparative effectiveness research: defining, reporting and interpreting nonrandomized studies of treatment effects using secondary data sources: the ISPOR Good Research Practices for Retrospective Database Analysis Task Force Report: part I. Value Health. 2009;12(8):1044-1052. doi:10.1111/j.1524-4733.2009.00600.x 19793072

[7] Fonager K, Sørensen HT, Rasmussen SN, Møller-Petersen J, Vyberg M. Assessment of the diagnoses of Crohn’s disease and ulcerative colitis in a Danish hospital information system. Scand J Gastroenterol. 1996;31(2):154-159. doi:10.3109/00365529609031980 8658038

[8] Rungoe C, Langholz E, Andersson M, . Changes in medical treatment and surgery rates in inflammatory bowel disease: a nationwide cohort study 1979-2011. Gut. 2014;63(10):1607-1616. doi:10.1136/gutjnl-2013-305607 24056767

[9] Nyboe Andersen N, Pasternak B, Friis-Møller N, Andersson M, Jess T. Association between tumour necrosis factor-α inhibitors and risk of serious infections in people with inflammatory bowel disease: nationwide Danish cohort study. BMJ. 2015;350:h2809. doi:10.1136/bmj.h2809 26048617 PMC4456959

[10] Nyboe Andersen N, Pasternak B, Basit S, . Association between tumor necrosis factor-α antagonists and risk of cancer in patients with inflammatory bowel disease. JAMA. 2014;311(23):2406-2413. doi:10.1001/jama.2014.5613 24938563

[11] Kirchgesner J, Nyboe Andersen N, Carrat F, Jess T, Beaugerie L; BERENICE study group. Risk of acute arterial events associated with treatment of inflammatory bowel diseases: nationwide French cohort study. Gut. 2020;69(5):852-858. doi:10.1136/gutjnl-2019-318932 31446428

[12] Kappelman MD, Horvath-Puho E, Sandler RS, . Thromboembolic risk among Danish children and adults with inflammatory bowel diseases: a population-based nationwide study. Gut. 2011;60(7):937-943. doi:10.1136/gut.2010.228585 21339206

[13] Charlson ME, Pompei P, Ales KL, MacKenzie CR. A new method of classifying prognostic comorbidity in longitudinal studies: development and validation. J Chronic Dis. 1987;40(5):373-383. doi:10.1016/0021-9681(87)90171-8 3558716

[14] Gilbert T, Neuburger J, Kraindler J, . Development and validation of a Hospital Frailty Risk Score focusing on older people in acute care settings using electronic hospital records: an observational study. Lancet. 2018;391(10132):1775-1782. doi:10.1016/S0140-6736(18)30668-8 29706364 PMC5946808

[15] Ertefaie A, Stephens DA. Comparing approaches to causal inference for longitudinal data: inverse probability weighting versus propensity scores. Int J Biostat. 2010;6(2):14. doi:10.2202/1557-4679.1198 21969998

[16] Austin PC. The use of propensity score methods with survival or time-to-event outcomes: reporting measures of effect similar to those used in randomized experiments. Stat Med. 2014;33(7):1242-1258. doi:10.1002/sim.5984 24122911 PMC4285179

[17] Lee SY, Wolfe RA. A simple test for independent censoring under the proportional hazards model. Biometrics. 1998;54(3):1176-1182. doi:10.2307/2533867 9840972

[18] Singh S, Murad MH, Fumery M, Dulai PS, Sandborn WJ. First- and second-line pharmacotherapies for patients with moderate to severely active ulcerative colitis: an updated network meta-analysis. Clin Gastroenterol Hepatol. 2020;18(10):2179-2191.e6. doi:10.1016/j.cgh.2020.01.008 31945470 PMC8022894

[19] Singh S, Murad MH, Fumery M, . Comparative efficacy and safety of biologic therapies for moderate-to-severe Crohn’s disease: a systematic review and network meta-analysis. Lancet Gastroenterol Hepatol. 2021;6(12):1002-1014. doi:10.1016/S2468-1253(21)00312-5 34688373 PMC8933137

[20] Kochar B, Pate V, Kappelman MD, . Vedolizumab Is associated with a lower risk of serious infections than anti-tumor necrosis factor agents in older adults. Clin Gastroenterol Hepatol. 2022;20(6):1299-1305. doi:10.1016/j.cgh.2021.08.047 34481954 PMC8891388

[21] Tsai L, Nguyen NH, Ma C, Prokop LJ, Sandborn WJ, Singh S. Systematic review and meta-analysis: risk of hospitalization in patients with ulcerative colitis and Crohn’s disease in population-based cohort studies. Dig Dis Sci. 2022;67(6):2451-2461. doi:10.1007/s10620-021-07200-1 34379220 PMC8831664

[22] Tsai L, Ma C, Dulai PS, . Contemporary Risk of Surgery in Patients With Ulcerative Colitis and Crohn’s Disease: A Meta-Analysis of Population-Based Cohorts. Clin Gastroenterol Hepatol. 2021;19(10):2031-2045. doi:10.1016/j.cgh.2020.10.039 33127595 PMC8934200

[23] Khan N, Pernes T, Weiss A, . Efficacy of vedolizumab in a nationwide cohort of elderly inflammatory bowel disease patients. Inflamm Bowel Dis. 2022;28(5):734-744. doi:10.1093/ibd/izab163 34245261

[24] Wyant T, Leach T, Sankoh S, . Vedolizumab affects antibody responses to immunisation selectively in the gastrointestinal tract: randomised controlled trial results. Gut. 2015;64(1):77-83. doi:10.1136/gutjnl-2014-307127 24763133

[25] Singh S, Heien HC, Herrin J, . Comparative risk of serious infections with tumor necrosis factor α antagonists vs vedolizumab in patients with inflammatory bowel diseases. Clin Gastroenterol Hepatol. 2022;20(2):e74-e88. doi:10.1016/j.cgh.2021.02.032 33640480 PMC8384969

[26] Lichtenstein GR, Feagan BG, Cohen RD, et al. Serious infection and mortality in patients with Crohn's disease: more than 5 years of follow-up in the TREAT registry. Am J Gastroenterol. 2012;107(9):1409-1422. doi:10.1038/ajg.2012.218 22890223 PMC3438468

[27] DʼHaens G, Reinisch W, Panaccione R, et al. Lymphoma risk and overall safety profile of adalimumab in patients with Crohn's disease with up to 6 years of follow-up in the Pyramid registry. Am J Gastroenterol. 2018;113(6):872-882. doi:10.1038/s41395-018-0098-4 29867173

[28] Asscher VER, Biemans VBC, Pierik MJ, ; Dutch Initiative on Crohn and Colitis (ICC). Comorbidity, not patient age, is associated with impaired safety outcomes in vedolizumab- and ustekinumab-treated patients with inflammatory bowel disease—a prospective multicentre cohort study. Aliment Pharmacol Ther. 2020;52(8):1366-1376. doi:10.1111/apt.16073 32901983 PMC7539998

[29] Kochar B, Cai W, Cagan A, Ananthakrishnan AN. Pretreatment frailty is independently associated with increased risk of infections after immunosuppression in patients with inflammatory bowel diseases. Gastroenterology. 2020;158(8):2104-2111. doi:10.1053/j.gastro.2020.02.032 32105728

[30] Singh S, Heien HC, Sangaralingham L, . Frailty and risk of serious infections in biologic-treated patients with inflammatory bowel diseases. Inflamm Bowel Dis. 2021;27(10):1626-1633. doi:10.1093/ibd/izaa327 33325507 PMC8522787

[31] Grijalva CG, Chung CP, Stein CM, . Computerized definitions showed high positive predictive values for identifying hospitalizations for congestive heart failure and selected infections in Medicaid enrollees with rheumatoid arthritis. Pharmacoepidemiol Drug Saf. 2008;17(9):890-895. doi:10.1002/pds.1625 18543352 PMC4861217

