# Supplementary material for: Comparative Outcomes and Safety of Vedolizumab vs Tumor Necrosis Factor Antagonists for Older Adults With Inflammatory Bowel Diseases
Source: JAMA Netw Open. 2022 Sep 30;5(9):e2234200. doi: 10.1001/jamanetworkopen.2022.34200 (PMC9526086; doi:10.1001/jamanetworkopen.2022.34200)
Supplement: Supplement. — eTable 1. Baseline Characteristics of Older Patients With IBD Treated With Vedolizumab vs TNF Antagonists Before Propensity Score Matching eTable 2. Comparative Effectiveness and Safety of Vedolizumab vs TNF Antagonists in Older Patients With IBD, Using Inverse Probability of Treatment Weighted Analysis eFigure. Standardized Mean Differences of Covariates Before and After Propensity Score Matching [file jamanetwopen-e2234200-s001.pdf]

Supplementary Online Content

Singh S, Iversen AT, Allin KH, Jess T. Comparative outcomes and safety of vedolizumab vs tumor necrosis factor antagonists for older adults with inflammatory bowel diseases. *JAMA Netw Open.* 2022;5(9):e2234200. doi:10.1001/jamanetworkopen.2022.34200

eTable 1. Baseline Characteristics of Older Patients With IBD Treated With Vedolizumab vs TNF Antagonists Before Propensity Score Matching
eTable 2. Comparative Effectiveness and Safety of Vedolizumab vs TNF Antagonists in Older Patients With IBD, Using Inverse Probability of Treatment Weighted Analysis
eFigure. Standardized Mean Differences of Covariates Before and After Propensity Score Matching

This supplementary material has been provided by the authors to give readers additional information about their work.

© 2022 Singh S et al. *JAMA Network Open.*

eTable 1. Baseline Characteristics of Older Patients With IBD Treated With Vedolizumab vs TNF Antagonists Before Propensity Score Matching

Baseline characteristics	Incident users of vedolizumab (n=379) (%)	Incident users of TNF antagonists (n=3,132) (%)
Age of patients		
• 50-60y	197 (52.0)	1674 (53.4)
• 61-70y	109 (28.8)	998 (31.9)
• >70y	73 (19.3)	460 (14.7)
Female, n (%)	204 (53.8)	1593 (50.9)
Area socioeconomic index		
• Quartile 1	96 (25.3)	796 (25.4)
• Quartile 2	96 (25.3)	808 (25.8)
• Quartile 3	118 (31.1)	772 (24.6)
• Quartile 4	69 (18.2)	756 (24.1)
IBD subtype		
• Crohn's disease	178 (47.0)	1499 (47.9)
• Ulcerative colitis	201 (53.0)	1633 (52.1)
Charlson comorbidity score		
• 0	249 (65.7)	2218 (70.8)
• 1	67 (17.7)	568 (18.1)
• 2 or more	63 (16.6)	346 (11.0)
Hospital frailty risk score		
• Low risk (<5)	342 (90.2)	2900 (92.6)
• Intermediate risk (5-15)	27 (7.1)	194 (6.2)
• High risk (>15)	10 (2.7)	38 (1.2)
**Disease characteristics**		
Prior IBD hospitalization within 1y prior to biologic initiation	139 (36.7)	1104 (35.2)
Prior IBD-related major surgery within 5y prior to biologic initiation	53 (14.0)	288 (9.2)
Prior IBD-related minor surgery within 5y prior to biologic initiation	20 (5.3)	210 (6.7)
Prior serious infection within 1y prior to biologic initiation	41 (10.8)	273 (8.8)
**Treatment characteristics**		
Concomitant immunomodulator use with biologic initiation	25 (6.6)	723 (23.2)
Concomitant corticosteroid use with biologic initiation	123 (32.5)	672 (21.6)
Prior azathioprine use, 0-6m prior to biologic initiation	57 (15.0)	912 (29.1)
Prior corticosteroid use, 0-6m prior to biologic initiation	174 (45.9)	1474 (47.1)
Prior TNF antagonist exposure, 0-12m prior to index biologic initiation<br>• None	110 (29.0)<br>42 (11.1)<br>227 (59.9)	2478 (79.3)<br>172 (5.5)<br>476 (15.2)

© 2022 Singh S et al. *JAMA Network Open.*

• Primary non-response to TNF antagonist<br>• Secondary loss of response to TNF antagonist		

*Patients could contribute to multiple different exposures, hence unit of analysis was patient-treatment episode
[Abbreviations: IBD=inflammatory bowel diseases; m=months; TNF=tumor necrosis factor; y=years]

© 2022 Singh S et al. *JAMA Network Open.*

eTable 2. Comparative Effectiveness and Safety of Vedolizumab vs TNF Antagonists in Older Patients With IBD, Using Inverse Probability of Treatment Weighted Analysis

Outcomes	**Adjusted HR (95% CI),<br>Vedolizumab vs. TNF antagonists**
Composite treatment failure	1.19 (0.96, 1.48)
• IBD-related hospitalization	1.24 (0.94, 1.65)
• Major abdominal surgery	1.79 (1.27, 2.52)
• New steroid use	1.21 (0.91, 1.60)
Serious infection	0.93 (0.59-1.48)

*Additionally adjusted for Charlson comorbidity and frailty
[Abbreviations: CI-confidence interval; HR-hazard ratio; IBD=inflammatory bowel diseases; TNF=tumor necrosis factor]

© 2022 Singh S et al. *JAMA Network Open.*

eFigure. Standardized Mean Differences of Covariates Before and After Propensity Score Matching

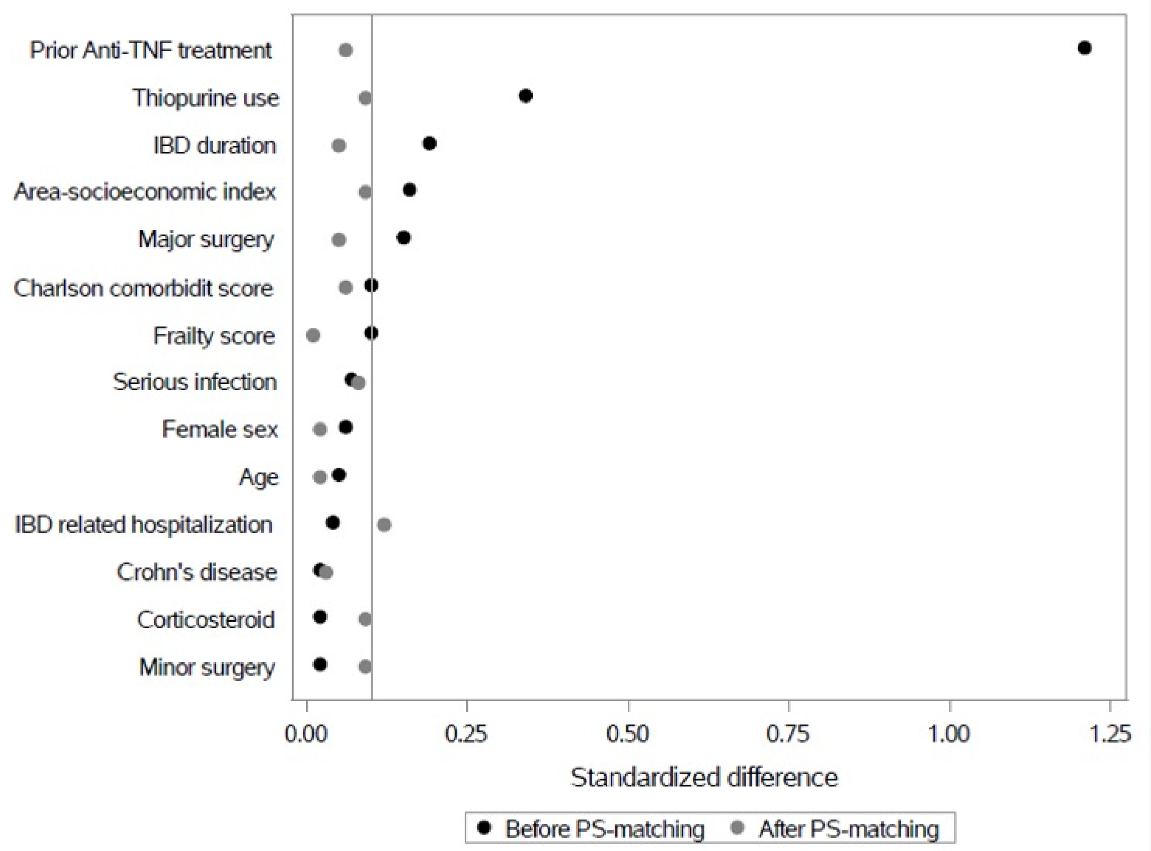

© 2022 Singh S et al. *JAMA Network Open.*
